# Therapy of Hypoparathyroidism by Replacement with Parathyroid Hormone

**DOI:** 10.1155/2014/765629

**Published:** 2014-07-01

**Authors:** Lars Rejnmark, Line Underbjerg, Tanja Sikjaer

**Affiliations:** Department of Endocrinology and Internal Medicine, Aarhus University Hospital, Tage-Hansens Gade 2, 8000 Aarhus, Denmark

## Abstract

Hypoparathyroidism (HypoPT) is a state of hypocalcemia due to inappropriate low levels of parathyroid hormone (PTH). HypoPT is normally treated by calcium supplements and activated vitamin D analogues. Although plasma calcium is normalized in response to conventional therapy, quality of life (QoL) seems impaired and patients are at increased risk of renal complications. A number of studies have suggested subcutaneous injections with PTH as an alternative therapy. By replacement with the missing hormone, urinary calcium may be lowered and QoL may improve. PTH replacement therapy (PTH-RT) possesses, nevertheless, a number of challenges. If PTH is injected only once a day, fluctuations in calcium levels may occur resulting in hypercalcemia in the hours following an injection. Twice-a-day injections seem to cause less fluctuation in plasma calcium but do stimulate bone turnover to above normal. Most recently, continuous delivery of PTH by pump has appeared as a feasible alternative to injections. Plasma calcium levels do not fluctuate, urinary calcium is lowered, and bone turnover is only stimulated modestly (into the normal range). Further studies are needed to assess the long-term effects. If beneficial, it seems likely that standard treatment of HypoPT in the future will change into replacement therapy with the missing hormone.

## 1. Introduction

Hypoparathyroidism (HypoPT) is diagnosed on the basis of hypocalcemia with low plasma levels of parathyroid hormone (PTH). In most instances, HypoPT is due to accidental damages to or removal of the parathyroid glands during neck surgery. More rare causes include genetic or autoimmune disorder such as DiGeorge (22q11.2 deletion) syndrome or hypoparathyroidism-retardation-dysmorphism syndrome. In addition, a state of hypoparathyroid hypocalcemia may be caused by an activating mutation in the calcium sensing receptor (autosomal dominant hypocalcemia) [1–3].

## 2. How Prevalent Is Hypoparathyroidism?

There are only few data available on the epidemiology of HypoPT. In a recent nationwide case-finding study by our group, we identified all patients diagnosed with postsurgical HypoPT in Denmark [4]. By reviewing individual patient charts, we found a total of 1849 patients with postsurgical HypoPT, among whom 1127 were alive at the date of follow-up. Our data suggest that the prevalence of postsurgical HypoPT in Denmark is 22/100,000 individuals. This is somehow in accordance with a preliminary report from the Mayo Clinic in which a total of 54 patients (71% women) with HypoPT were identified. Among cases, 78% were classified as postsurgical, suggesting a prevalence of postsurgical HypoPT of approximately 29 in 100,000 [5]. Most recently, the number of insured adult patients with HypoPT in the United States has been estimated to be approximately 58,700 individuals [6]. Assuming that approximately 15% of US population is without health insurance and a total US population of 313 mio. persons (US citizens), the prevalence of chronic HypoPT within the insured population (266 mio.) in US is 22/100,000 which is in full agreement with the estimate from our study.

## 3. Is Comorbidity Increased in Patients with Hypoparathyroidism?

HypoPT is associated with neuromuscular complaints, a decreased quality of life (QoL), and an increased risk of neuropsychiatric disorders [7–10]. The reason for this has so far not been fully understood. Calcium is of importance to cellular processes throughout the organism. A tight regulation of plasma calcium is needed, as calcium ions have a stabilizing effect on voltage-gated ion channels. If plasma calcium levels are too low, voltage-gated calcium channels start opening spontaneously, causing an increased neuromuscular irritability, whereas hypercalcemia causes a depressed function of the neuromuscular system. Although treatment of HypoPT aims at normalizing plasma calcium levels, the treatments available do not fully restore calcium homeostasis, as unphysiological fluctuations may occur, as well as the minute-to-minute regulations of plasma calcium levels being not fully restored although calcium levels, on average, may be (near) normal. Moreover, receptors for PTH are expressed by cells in several tissues including the central nervous system [11]. Specific effects of PTH beyond the well-known effects on calcium homeostasis and bone metabolism need further elucidations, but an increasing number of studies do suggest such effects. For example, an effect of PTH on the adrenal cortex has recently been discovered [12]. If PTH also affects cells in the central nervous system, this may contribute to the impaired QoL and the increased risk of neuropsychiatric diseases in HypoPT.

In the recently published study on the epidemiology of HypoPT by our group, we investigated risk of renal complication, cardiovascular diseases, and mortality [4]. Compared with a group of age- and gender-matched population based controls, our study showed an almost four-time increased risk of renal complications in patients with HypoPT. This was true for renal stone diseases as well as for risk of renal insufficiency. As PTH normally increases the renal excretion of phosphate, the calcium-phosphate product may be elevated in patients with HypoPT causing an increased risk of extraskeletal precipitation of calcium-phosphate crystals [13, 14]. In patients with long-standing HypoPT (including inherited forms), an increased risk of calcium deposits in the basal ganglia has also been reported [15, 16]. However, patients with HypoPT have not been seen to have an increased risk of arteriosclerotic vascular disease, as our study showed an incidence of cardiovascular diseases similar to the general background population. Moreover, we did not find evidence of an altered mortality in HypoPT [4].

## 4. Conventional Therapy of Hypoparathyroidism

The mainstay of conventional treatment of HypoPT is calcium supplement in combination with vitamin D. Before the development of activated vitamin D analogues in terms of either 1*α*-hydroxylated vitamin D (alfacalcidol) or 1,25-dihydroxyvitamin D (calcitriol), patients with HypoPT were treated with native vitamin D analogues such as cholecalciferol (vitamin D3) or ergocalciferol (vitamin D2).

If treated with native vitamin D, very high doses are normally needed to achieve normocalcemia resulting in markedly elevated plasma levels of 25-hydroxyvitamin D (25OHD). Typically, patients receive a daily dose of 50,000 to 200,000 IU of vitamin D2 or D3 and plasma 25OHD concentrations are in the range of 500–1000 nmol/l [3]. The rationale for this is that although the affinity of 1,25(OH)_2_D to the vitamin D receptor (VDR) is much higher than the affinity of native vitamin D analogues, these nonhydroxylated vitamin D metabolites do, nevertheless, bind to the VDR and exert biological effects if their concentrations are high enough. Moreover, although the 1*α*-hydroxylase does not function properly if PTH is missing, a certain (small) amount of 25OHD is still converted into its active metabolite [3]. Since the development of 1*α*-hydroxylated vitamin D analogues, most patients with HypoPT have been shifted to treatment with either alfacalcidol or calcitriol, as the plasma half-life of activated vitamin D metabolites is much shorter (approximately 3–6 hours) than the biological half-life of vitamin D2 or D3 (approximately 3 weeks) [3]. The advantage of the shorter plasma half-life of activated vitamin D metabolites is that a new equilibrium is obtained at a much faster rate. Dose titration may be performed every 2-3 days if activated vitamin D metabolites are used, whereas 2-3 months have to pass before a new equilibrium is obtained in response to dose titration of native vitamin D analogues. Similarly, if intoxication occurs, this may have a much longer duration if caused by too high levels of native vitamin D compared with too high levels of calcitriol.

Different traditions seem to exist on how the relative dose of calcium and vitamin D is titrated. At some institutions, a relative high dose of calcium (3–5 gram/d) is combined with a relative low dose of activated vitamin D, whereas other institutions prefer to treat HypoPT with a lower dose of calcium supplements (800–1200 mg/d) with a relative high dose of activated vitamin D analogues. No formal comparison of these two treatment regimens has so far been performed. However, as lack of PTH causes too low 1,25(OH)_2_D concentrations to maintain a sufficient intestinal calcium absorption, it seems somehow more reasonable to correct the deficiency in activated vitamin D rather than compensating for 1,25(OH)_2_D deficiency by providing large amounts of calcium from supplements.

The therapeutic aim of the treatment is to obtain normocalcemia and to reduce long-term complications. If tolerated by the patient without symptoms of hypocalcemia, plasma calcium levels should be in the lower range (or slightly below the lower limit) of the reference interval. The reason for this is to avoid a too high renal calcium excretion and to lower the calcium-phosphate product. Due to the lack of PTH, urinary calcium levels are often markedly elevated in HypoPT which may predispose to the development of urolithiasis in a similar manner, as the increased risk in patients with hypercalciuria due to other etiologies [19]. If renal calcium excretion is high, thiazide diuretics may be used to lower urinary calcium. An elevated calcium-phosphate product may predispose to precipitation of calcium-phosphate salts causing extraskeletal calcifications. If possible, calcium-phosphate product should be below 4.4 mmol^2^/L^2^.

## 5. PTH Replacement Therapy (PTH-RT)

As detailed above, patients with HypoPT often have a number of complaints despite (near-)normal plasma calcium levels in response to conventional treatment [7–10]. HypoPT is one of the only hormonal insufficiency states which are not treated by replacement with the missing hormone. Almost 100 years ago, Fuller Albright showed that bovine extract of PTH was able to abolish symptomatic hypocalcemia [21]. A second paper was published in 1967, showing similar positive effects in two patients with HypoPT [22]. However, the use of bovine PTH extract caused development of neutralizing antibodies which limited the efficacy of bovine PTH, and no further studies were performed on PTH-RT before it became possible to produce PTH by recombinant DNA technology in the nineties. 10 years ago, recombinant human PTH (rhPTH) was marketed for the treatment of osteoporosis. PTH is an 84 amino acids polypeptide hormone. Apparently, the N-terminal part is the biological active fragment of the molecule. In addition to the intact PTH_1–84_ molecule, the N-terminal part of the molecule (PTH_1–34_, named teriparatide) has also been developed. In a number of NIH-sponsored randomized studies, effects of PTH_1–34_RT have been investigated by a group led by Karen Winer in Maryland, USA [23–28]. In a recently published study by our group, we investigated effects of PTH_1–84_RT [17, 18, 20]. In addition, data on effects of PTH_1–84_RT in HypoPT are available from an open-label nonrandomized cohort study performed by Columbia University, New York, USA [29], and from a recently published randomized controlled study investigating effects of different doses of PTH_1–84_ (the REPLACE study) [30].

### 5.1. Effects of PTH_1–34_RT in HypoPT

In the studies by Winer et al., it was shown that normocalcemia can be obtained in patients with HypoPT by PTH-RT. In the studies, dose of PTH_1–34_ was carefully titrated in such a manner that plasma calcium levels could be maintained within the lower part of the reference range or slightly below without any needs for concomitant therapy with activated vitamin D analogues. Due to the nature of the PTH molecule (polypeptide), the drug needs to be injected subcutaneously, as it is broken down if administrated orally. In adults as well as in children, Winer et al. [23, 26] showed that administration of PTH_1–34_ injections twice a day was better than once-a-day injections. Although mean 24 h plasma calcium levels did not differ significantly between once-a-day injections as compared with twice-a-day injections, the 24 h plasma calcium profile was smoother if PTH_1–34_ was given twice a day. Compared with twice-a-day administration, one daily injection resulted in significantly higher plasma calcium levels during the first part of the day with significant lower levels during the second half of the day [23, 26]. Moreover, daily dose of PTH needed to maintain normocalcemia was lower with twice-a-day injections and the patients preferred to inject themselves twice a day which may be due to less fluctuation in plasma calcium levels. In the most recent study by Winer et al. [25], PTH_1–34_ was administrated by pump delivery which resulted in an even smoother 24 h plasma calcium profile than twice-a-day injections and further reduced total daily dose of PTH_1–34_ needed to maintain normocalcemia.

### 5.2. Effects of PTH_1–84_RT on Calcium Homeostasis in HypoPT

**Figure 1:**
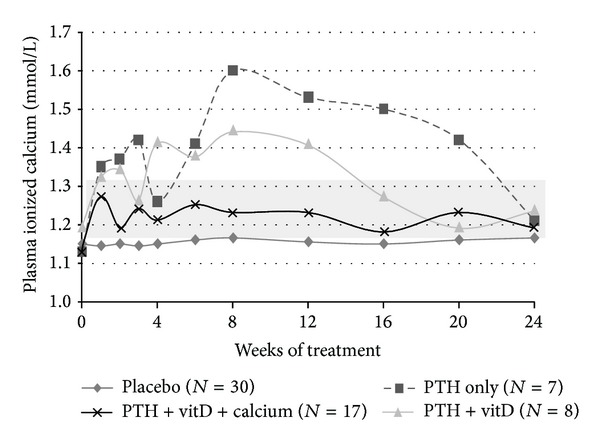
Results from a randomized controlled study on effects of PTH_1–84_ replacement therapy versus conventional treatment on plasma ionized calcium levels. Patients in the placebo group received placebo injections in combination with conventional therapy (calcium supplements and activated vitamin D analogues). Data are stratified by whether patients at the end of study were managed on treatment with PTH alone (PTH only), PTH plus activated vitamin D (PTH + VitD), PTH plus activated vitamin D and calcium supplements (PTH + VitD + calcium), or placebo. The background light gray square indicates the reference range (1.18–1.32 mmol/l). Reproduced with permission from JBMR [17].

Although the N-terminal part of the PTH molecule is considered as the active part, the C-terminal part of the hormone may also affect signaling pathways, although the precise effects of the C-terminal part have so far not been well characterized [31]. Moreover, PTH_1–34_ and PTH_1–84_ have different pharmacokinetic and pharmacodynamic profiles (Table 1) why the effect of treatment with the two drugs may differ. Accordingly, we undertook a randomized controlled trial in order to assess whether PTH_1–84_ is feasible for replacement therapy. In our study, 62 patients with long-standing HypoPT were randomized to a daily injection with PTH_1–84_ or similar placebo in a double-blind manner. In contrast to the study design used by Winer et al., we administered PTH_1–84_ in a fixed daily dose of 100 *μ*g as an add-on to conventional therapy. Accordingly, our study patients were throughout our trial followed up at our out-patient clinic, whereas the patients studied by Winer et al. were admitted to a two-week hospital stay in order to titrate dose of PTH. In our study, daily dose of calcium and alfacalcidol/calcitriol was down-titrated if plasma calcium levels increased in response to the injections. In accordance with the findings by Winer et al., we also showed that needs for calcium supplements and activated vitamin D analogues were reduced in response to PTH-RT with PTH_1–84_. Following six months of PTH_1–84_RT, the median dose of alfacalcidol/calcitriol decreased by 50% in response to PTH_1–84_ with no changes in the placebo group. Seven patients in the PTH group stopped treatment with activated vitamin D completely with none in the placebo arm (*P* < 0.01). Furthermore, use of oral calcium supplements was stopped in 15 patients in the PTH group compared with none in the placebo group (*P* < 0.01). Despite a complete stop of calcium supplements and activated vitamin D analogues, five patients had sustained hypercalcemia as measured 24 h after the last injection with PTH_1–84_. In these five patients, PTH dose needed to be reduced to less than one daily injection of 100 *μ*g. At end of study, they received PTH injections either five times a week (*n* = 3), every second day (*n* = 1), or every third day (*n* = 1).  Figure 1 shows plasma ionized calcium levels during the 24 weeks of trial, as measured 24 h after last injection and stratified by whether studied patients at end of trial were managed by monotherapy with PTH_1–84_ or in addition to PTH_1–84_ also needed calcium supplements and/or alfacalcidol in order to maintain normocalcemia. Importantly, although most patients randomized to PTH_1–84_RT developed hypercalcemia during the first part of the trial (before downtitration of conventional treatment), studied patients had normocalcemia at end of trial. However, as shown in Figure 1, most patients did need alfacalcidol (in a reduced dose compared with prior to initiation of PTH_1–84_RT) in order to maintain normocalcemia 24 h following the last injection, although 7 patients were treated with PTH_1–84_ alone. Accordingly, similar to conventional treatment, replacement therapy with PTH requires individual dosing; one dose does not fit all!

**Figure 2:**
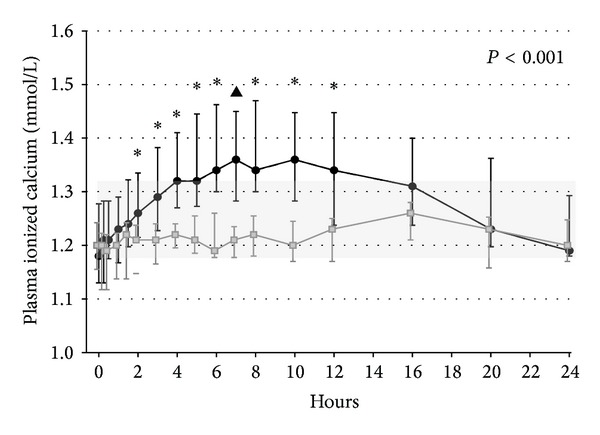
Diurnal variations in plasma ionized calcium levels in patients with hypoparathyroidism following 24 weeks of treatment with 100 *μ*g of PTH_1–84_ (black) (*n* = 21) or placebo (gray) (*n* = 17). The background light gray square indicates the reference range (1.18–1.32 mmol/l). The *P* value indicates significance between groups by repeated measurements ANOVA. Median with interquartile (25% to 75% percentile) range. ^▲^ *P* < 0.01 and ^∗^ *P* < 0.001 by post hoc comparison. Reproduced with permission from JBMR [18].

In addition to measurement of nadir plasma calcium levels (obtained 24 h after last injection), we also performed a study on diurnal variation of plasma calcium levels at the end of our trial. As shown in Figure 2, injections with PTH_1–84_ caused a marked diurnal variation in plasma calcium levels with a marked rise in calcium levels during the first 6–8 hours after the injection followed by a decrease towards starting levels during the rest of the 24 h study period. In the PTH_1–84_ group, 71% of the patients had one or more measurements of plasma calcium levels above the upper limit of the reference interval during the 24 h study. These findings underline the fact that measurements of nadir levels of plasma calcium do not provide a full picture of diurnal changes in calcium levels during the day. In the most recent study on PTH_1–84_RT (the REPLACE study), dose titration was made possible by administrating PTH_1–84_ once a day in a fixed dose of either 50 *μ*g, 75 *μ*g, or 100 *μ*g [30]. Similar to our findings, this study showed a significant reduction in needs for calcium supplements and activated vitamin D analogues. Unfortunately, no data on the 24 h profile of plasma calcium levels following injections with PTH_1–84_ is available from this study. Most likely, a lower dose than 100 *μ*g causes less hypercalcemia in the hours following the injection. However, it seems likely that diurnal variations in plasma calcium levels occur in response to PTH_1–84_ injections even if a lower dose is administered.

### 5.3. Effects on Urinary Calcium

**Figure 3:**
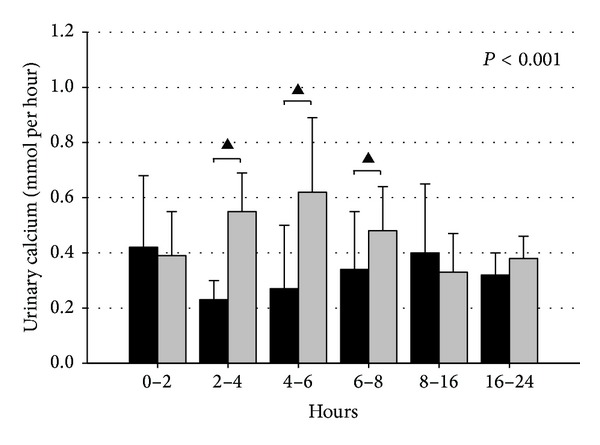
Diurnal variations in renal excretion of calcium (excretion per hour) in patients with hypoparathyroidism following 24 weeks of treatment with 100 *μ*g of PTH_1–84_ (black) (*n* = 21) or placebo (gray) (*n* = 17). *P* value indicates significance between group differences by repeated measurements ANOVA. ^▲^ *P* < 0.01 by post hoc comparison. Reproduced with permission from JBMR [18].

In the studies by Winer et al. [27] on PTH_1–34_RT, a tendency towards a reduced renal calcium excretion was found with a normalization of 24 h urinary calcium during long-term therapy with two daily injections. Interestingly, compared with two daily injections of PTH_1–34_, pump delivery of PTH_1–34_ caused a significantly reduced renal calcium excretion [25]. Urinary calcium was not reduced in our randomized controlled trial or in the REPLACE study in response to PTH_1–84_ injected once a day [30]. Our study on the diurnal profile of renal calcium excretion following an injection with PTH_1–84_ showed a reduced renal calcium excretion from 2 to 8 hours following the injection (Figure 3). This is in accordance with the pharmacodynamic characteristics of PTH_1–84_, that is, that plasma levels peak after 1-2 h with a plasma half-life of approximately 1.5 h (Table 1). Accordingly, PTH-RT does reduce urinary calcium (by increasing the renal tubular reabsorption) as long as PTH is present in the circulation. However, once-a-day injection does not provide a sustained* exposure* of PTH to the renal tubules throughout the day and no marked net effect on 24 h urinary calcium is therefore present. Moreover, the rise in plasma calcium levels in the hours following an injection with PTH increased the filtered load of calcium and may thereby in part antagonize the net effect on urinary calcium as induced by a PTH mediated increased renal tubular calcium reabsorption. Moreover, PTH-RT causes an increase in the synthesis of 1,25(OH)_2_D with a peak concentration approximately 10 h after an injection [18]. This may contribute to the lack of an effect of once-daily PTH injections on the 24 h urinary calcium excretion, as 1,25(OH)_2_D might maintain plasma calcium during the second half of the day, while causing an increase in urinary calcium excretion.

## 6. Effects of PTH-RT on Bone

Due to lack of PTH, HypoPT is a state of low bone turnover with highly (over-)mineralized bone. In all published studies on bone effects of PTH-RT, administration of PTH has caused a marked increase in bone turnover as assessed by biochemical bone turnover markers (BTM) and histomorphometric analyses of bone biopsies [29]. In the study by Winer et al. [27], levels of BTM increased for the first 2.5 years of treatment. The total duration of the study was three years. During the last 6 months of study, levels of BTM seemed to decrease although levels at the end of study were significantly increased compared with baseline values as well as compared with the upper limit of the reference interval. Recently, Cusano et al. [33] reported data from a 4-year cohort study on PTH_1–84_RT in which studied patients received a fixed dose of PTH_1–84_ 100 *μ*g every other day. Following an initial rise in levels of BTM during the first year of trial, levels decreased and were within the reference interval at end of study. Our study lasted only 6 months. Similar to other published studies, BTM in our patients treated with PTH_1–84_ increased markedly. Interestingly, in the recently published study on pump delivery of PTH_1–34_, markers did increase in response to pump delivery but stayed within the reference range and the increase in BTM was less pronounced than the increase in response to injection with PTH_1–34_ twice a day.

**Figure 4:**
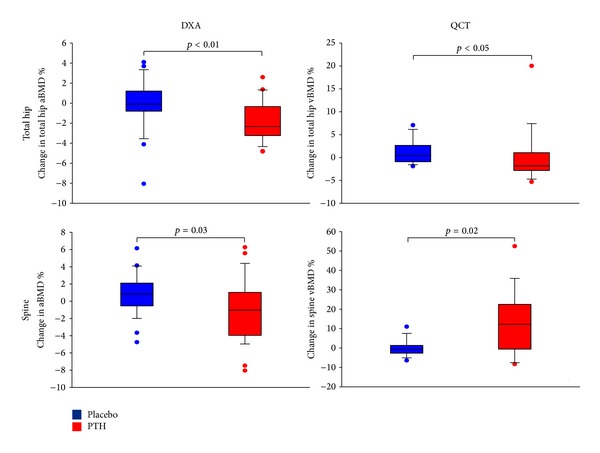
Changes in areal bone mineral density (aBMD) and volumetric bone mineral density (vBMD) as assessed by DXA and QCT scans, respectively. Changes were assessed between baseline and 24 weeks of trial during which studied patients were treated with 100 *μ*g of PTH_1–84_ (read) or placebo (blue) [20].

**Figure 5:**
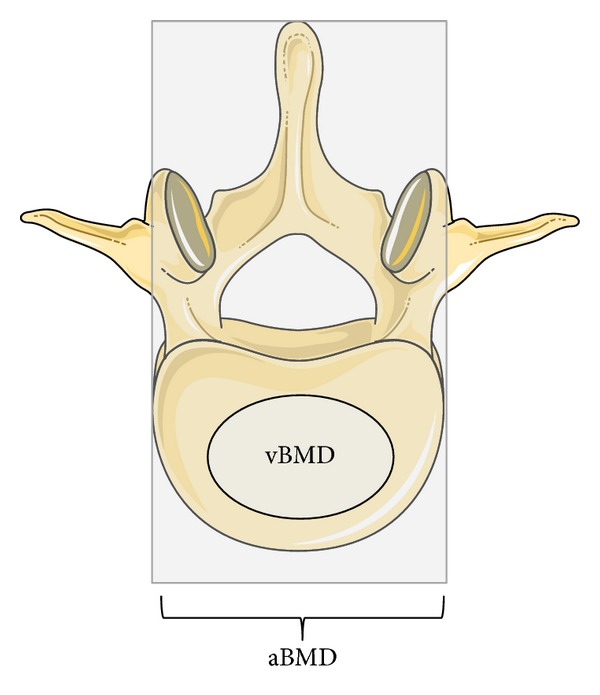
Regions of the lumbar spine vertebra included in measurement of bone mineral density by DXA and QCT scans. In addition to the vertebral body, areal bone mineral density (aBMD) as assessed by DXA includes density measurement of the posterior processes (spinous process) as indicated by the background light gray square. Measurement of volumetric bone mineral density (vBMD) by QCT-scans includes only the central (trabecular) part of the vertebral body.

In accordance with the increased bone turnover, our study showed a decreased bone mineral density (BMD) in response to PTH_1–84_RT as assessed by DXA scans at the lumbar spine, hip, and whole body. [17]. However, additional assessment of 3-dimensional volumetric bone mineral density (vBMD) by quantitative computer tomography (QCT) showed a significantly increased vBMD at the trabecular compartment of the lumbar spine (Figure 4) [20]. Importantly, BMD as measured by DXA is a 2-dimensional assessment of areal BMD (aBMD). In the anterior-posterior projection, this included BMD of the posterior processes which are composed of mainly cortical bone. Accordingly, aBMD of the lumbar spine is a composite measure of bone density in cortical and trabecular bone whereas vBMD at the spine as measured by QCT only includes the trabecular bone compartment (Figure 5). Although the lumbar spine is considered as a skeletal site that contains mostly trabecular bone (compared with, e.g., the hip and whole body), a large proportion (≈75%) of the aBMD measure of the vertebral body is, nevertheless, composited by cortical bone [34]. In osteoporosis, therapy with PTH in terms of one daily injection has been marketed as a bone anabolic treatment as it causes bone anabolic effects on trabecular bone. In cortical bone, however, PTH_1–84_ has been reported to cause a tendency towards an increased cortical porosity [35]. As bone turnover in HypoPT, prior to initiation of PTH-RT, is abnormally low, the effect on cortical bone, in terms of an increased remodeling activity causing an increased cortical porosity, is most likely more pronounced than in osteoporotic patients with a normal (or elevated) bone turnover prior to PTH therapy. The balance between anabolic effects on trabecular bone and catabolic effects on cortical bone is most likely dose dependent, as injections with PTH_1–84_ in a dose of 100 *μ*g every other day have been reported to cause a continuous increase in aBMD at the lumbar spine with no major effects at other skeletal sites during four years of treatment [33].

Overall, PTH-RT reverses the abnormal low bone turnover with bone anabolic effects on trabecular bone. The increased bone turnover may be of advantages, but an anabolic effect on trabecular bone is not necessarily needed, as BMD in HypoPT is relatively high if previously treated with conventional therapy. Data on BTM in response to pump delivery suggest a more modest increase compared with the increase if PTH is administered as once- or twice-a-day injections. Accordingly, continuous delivery of PTH seems to normalize bone turnover in a more physiological manner than therapy with injections.

## 7. Effects of PTH-RT Beyond Calcium Homeostasis and Bone

Winer at al. [27] reported that their patients preferred PTH_1–34_RT due to a feeling of an improved well-being but provided no formal data on QoL. In the open-label cohort study on injections with PTH_1–84_ 100 *μ*g every other day, Cusano el al. [36] have recently reported a beneficial effect on QoL in terms of an improved physical and mental functioning as assessed by the RAND36-Item Health Survey. However, as the trial did not include a control group, the study does not allow for causal conclusions on effects of PTH-RT on QoL. Similarly, no data are available on long-term outcomes of PTH-RT such as risk of fracture or renal complications.

## 8. Perspective

HypoPT is one of the only hormonal insufficiency states not treated by substitution with the missing hormone. In recent years, PTH-RT has in a number of studies been shown to be an alternative to conventional treatment. PTH may abolish or reduce the need for calcium supplements and activated vitamin D analogues while maintaining eucalcemia. Available data do suggest an improved QoL in response to PTH-RT, but further studies are needed to establish this as well as long-term effects on bone and nonskeletal health. Conventional treatment is associated with an increased risk of renal complications, including nephrolithiasis and impairment of renal function. As PTH increases the renal tubular reabsorption of calcium, PTH-RT may lower urinary calcium and thereby protect against renal calcifications. The optimal treatment regime needs, however, further clarifications. Injections with PTH once or twice a day have not been shown consistently to lower urinary calcium and intermittent hypercalcemia may develop in the hours following an injection. According to the most recent studies on PTH-RT, delivery by pump seems to be superior to injection therapy. By providing PTH-RT as a continuous infusion, plasma calcium levels do not fluctuate to any major degree and urinary calcium is reduced. Moreover, this form for delivery seems to cause a more gentle effect on bone turnover which is mildly increased into the normal range. Long-term effects need to be determined in further studies, as well as whether multiple daily injections with a low dose of PTH may be as feasible as pump delivery. If so, standard therapy of HypoPT may change from treatment with calcium and vitamin D into replacement therapy with the missing hormone.

## Figures and Tables

**Table 1:** Pharmacokinetic and -dynamic characteristics of recombinant human intact parathyroid hormone (rhPTH_1–84_) and teriparatide (rhPTH_1–34_) following a subcutaneous injection into the abdominal skin [32].

	PTH analogue	
	rhPTH_1 –34_	rhPTH_1 –84_
Bioavailability (%)	95%	55%
Time to peak PTH level (hours)	0.5 h	1-2 h
Plasma half-life (hours)	1.0 h	1.5 h
Time to peak plasma calcium levels (hours)	4–6 h	6–8 h

The data in the table are derived from EMEA online (http://www.ema.europa.eu/ema/). EMA Preotact product description and EMA Forsteo product description.
